# Skeletal muscle measures and physical function in older adults with cancer: sarcopenia or myopenia?

**DOI:** 10.18632/oncotarget.16866

**Published:** 2017-04-05

**Authors:** Grant R. Williams, Allison M. Deal, Hyman B. Muss, Marc S. Weinberg, Hanna K. Sanoff, Kirsten A. Nyrop, Mackenzi Pergolotti, Shlomit Strulov Shachar

**Affiliations:** ^1^ The University of Alabama at Birmingham, Birmingham, AL, USA; ^2^ The University of North Carolina at Chapel Hill, Chapel Hill, NC, USA; ^3^ UNC Lineberger Comprehensive Cancer Center, Chapel Hill, NC, USA; ^4^ Colorado State University, Fort Collins, CO, USA; ^5^ Division of Oncology, Rambam Health Care Campus, Haifa, Israel

**Keywords:** sarcopenia, myopenia, skeletal muscle index, cancer, geriatric oncology

## Abstract

**Background:**

Skeletal muscle loss, commonly known as sarcopenia, is highly prevalent in older adults and linked with adverse outcomes in cancer, yet the definition and role of sarcopenia remains uncertain. The aim of this study was to examine the association of Computerized Tomography (CT) assessed skeletal muscle measures with physical function in older adults with cancer.

**Results:**

CTs for 185 patients were available. Median age 73 (IQR 68–76) and 56.5% female. After controlling for sex and BMI, we found no evidence that SMI was associated with physical function impairments. Both SMD and SMG were associated physical function impairments and higher values were associated with decreased limitations in instrumental activities of daily living (RR 0.84 [CI 0.73–0.96] and 0.94 [CI 0.89–0.99], respectively), climbing stairs (RR 0.84 [CI 0.76–0.94] and 0.91 [CI 0.87–0.96]), walking 1 block (RR 0.77 [CI 0.67–0.90] and 0.91 [CI 0.85–0.97]), and prolonged Timed Up and Go (RR 0.83 [CI 0.75–0.92] and 0.92 [CI 0.88–0.96]).

**Materials and Methods:**

Using the Carolina Senior Registry, we identified patients with CT imaging performed within 60 days +/− of baseline geriatric assessment (GA). Skeletal muscle area and density (SMD) were analyzed from L3 lumbar segments. Muscle area and height (m^2^) were used to calculate skeletal muscle index (SMI). Skeletal Muscle Gauge (SMG) was created by multiplying SMI x SMD.

**Conclusions:**

Skeletal muscle mass as assessed from CT imaging was not associated with physical function impairments. Skeletal muscle radiodensity was more associated with physical function and may aid in identifying older adults at risk for functional impairments.

## INTRODUCTION

Skeletal muscle depletion, commonly known as sarcopenia within oncology, is highly prevalent in older adults with cancer and has been linked to increased toxicity and mortality in a variety of cancer types [1, 2]. Body composition analysis is not yet a standard part of the assessment of adults with cancer; however, computerized tomography (CT) images obtained as part of routine cancer staging and disease assessment are readily available tools that represent a nascent opportunity to provide additional prognostic information, particularly in older adults. Consistent methods for utilizing CT imaging to achieve practical and precise measurements of body composition exist and can be performed clinically without significant resource allocation [3]. These methods allow for practical CT-based measurements of skeletal muscle mass and radiodensity, as well as subcutaneous and visceral adiposity.

The precise definition of sarcopenia remains controversial. The term sarcopenia was first popularized in a study by Baumgartner *et al*. to describe age-related muscle mass loss seen in older adults in New Mexico [4]. Using dual-energy X-ray absorptiometry (DEXA), sarcopenia was defined as two standard deviations below the mean muscle mass of healthy younger adults and was shown to be highly prevalent in older adults. Ever since the term sarcopenia was introduced, numerous definitions have subsequently been used in the literature. A recent meta-analysis examining the prognostic value of sarcopenia in adults with solid tumors found 11 different definitions of sarcopenia in the included 38 studies [1]. The prevalence of sarcopenia using these various cut-points was wide ranging, from 11–74% depending on the study population and definition employed. These various definitions used in oncology based solely on low skeletal muscle mass, typically derived from optimum stratification analyses based on increased mortality or other adverse outcomes, limit the generalizability of studies and our understanding of study results.

Due to these challenges, a Sarcopenia Working Group was formed in 2009 to develop operational definitions and diagnostic criteria of sarcopenia, and ultimately recommended using both the presence of low muscle mass as well as evidence of low muscle strength or physical performance for the diagnosis of sarcopenia [5]. This two-part criterion for defining sarcopenia was suggested because the relationship between muscle strength and mass is not linear and muscle strength does not depend solely on muscle mass [6, 7]. Other important factors, such as muscle composition and fatty infiltration of muscle, complicate the use of muscle mass alone in defining sarcopenia. The working group also developed conceptual stages of sarcopenia that include pre-sarcopenia (low muscle mass alone), sarcopenia (low muscle mass with either reduced muscle strength or impaired physical performance), and severe sarcopenia (low muscle mass, reduced muscle strength, and impaired physical performance) [5]. Additional proposed clinical criteria for diagnosing sarcopenia developed since that time also highlight the importance of muscle weakness and poor physical function, and explicitly recommend against the use of reduced muscle mass alone [8, 9]. Furthermore, other terms such as myopenia and muscle wasting diseases have been developed and are suggested for use when describing clinically relevant muscle wasting that is not associated with loss of muscle strength or poor physical performance [10, 11].

Despite these guidelines, studies across the oncology field only utilize CT-based definitions of sarcopenia and often lack collaborative information on muscle strength or physical function. Therefore, we sought to explore the association of single-slice CT-based skeletal muscle measurements with physical function in older adults (age ≥ 65) with cancer. We hypothesized that skeletal muscle mass as measured by single-slice CT-imaging would be associated with physical performance and physical impairments.

## RESULTS

### Study population

For the 185 patients included in this study, mean age was 73 (standard deviation 6.8 years), 57% female, and 84% Caucasian (See Table 1). Most common cancer types included breast cancer (30%), lung cancer (20%), gastrointestinal malignancies (14%), and genitourinary malignancies (11%). The median time between the receipt of CT imaging and GA performance was 2 days (interquartile range of −16 to 0), and 83% of patients had imaging and the GA performed within 30 days of each other. Thirty-one percent of patients had the GA performed before cancer treatment was initiated and 51% during treatment. Forty-two percent of the sample had an impairment in IADL, 25% with one or more falls, and 44% with a prolonged TUG.

**Table 1:** Patient characteristics

	Total Sample (*n* = 185)
**Age, mean (range, SD)**	73 (65–93, 6.8)
**Gender, *n*(%)**	
Male	80 (43)
Females	105 (57)
**Race, *n* (%)**	
Caucasian	155 (84)
Black	28 (15)
Other	2 (1)
**Cancer type, *n* (%)**	
Breast cancer	53 (30)
Lung cancer	35 (20)
GI Malignancy	25 (14)
GU Malignancy	20 (11)
Heme Malignancy	16 (9)
Other	30 (17)
**Treatment Phase, *n* (%)**	
Before Treatment	56 (31)
During Treatment	90 (51)
After Treatment	33 (18)
**Education, *n* (%)**	
Some high school	24 (15)
High school degree	75 (46)
Associates/Bachelor's degree	35 (21)
Advanced degree	29 (18)
**Geriatric Assessment, *n* (%)**	
KPS < 80	43 (27)
1 or more falls	40 (25)
Prolonged TUG	81 (44)
IADL Impairment	64 (42)
Impaired bathing or dressing	27 (17)
Limited moderate activities	78 (48)
Limited climbing 1 flight of stairs	57 (36)
Limited walking 1 block	48 (30)
Limited bending, kneeling, or stooping	76 (47)

Abbreviations: GI, gastrointestinal; GU, genitourinary; KPS, Karnofsky Performance Status; TUG, Timed Up and Go; IADL, Instrumental Activities of Daily Living.

### Body composition metrics

Patients exhibited a wide variation in body composition. The average BMI was 27.1 (range 15.4–51.6), SMI 41.8 cm^2^/m^2^ (range 23–67), SMD 26.2 HU (range 3.9–47), and SMG 1103 AU (range 89–2760). Significant differences in SMI and SMG were found between men and women (45.7 versus 38.8 cm^2^/m^2^, *p* < 0.001 and 1244 versus 1000 AU, *p* < 0.001, respectively), while there was no differences in SMD or BMI by sex.

### Physical function and body composition correlations

No significant association of SMI with any measure of impaired physical function was found (See Table 2). Significant decreases in risk of impairment were found for increasing levels of both SMD and SMG with prolonged TUG, impaired IADL, climbing 1 flight of stairs, walking 1 block, and bending, kneeling, or stooping. For example, for each 5 unit increase in SMD, a patient's risk of having a prolonged TUG decreased by 17%. For each 100 unit increase in SMG, the risk of prolonged TUG decreased by 8%. Increasing SMD, but not SMG, was also found to be associated with lower risk of limitations in moderate activities. There was no significant association with low KPS, the presence of falls, or impaired bathing/dressing with any skeletal muscle metric. Even after additionally controlling for age and BMI in a secondary model, SMD and SMG remained significantly associated with physical function impairments, with the exception of the loss of significance with IADL for both. The model fit was similar for both models (with and without controlling for age and BMI), and overall AUCs ranged from 0.6 to 0.7. Furthermore, no differences in RR were found after controlling for treatment stage. Using the most commonly employed cut-point of SMI to define sarcopenia [12], we found no statistically significant differences in physical function impairments between sarcopenic and non-sarcopenic patients (data not shown).

**Table 2:** Relative risk of impairments in physical function by skeletal muscle measures after controlling for sex (*N* = 185)

	KPS < 80	≥ 1 falls	Prolonged TUG	Impaired IADL	Impaired Bathing and dressing	Limited Moderate activities	Limited in climbing 1 flight of stairs	Limited in walking 1 block	Limited in bending, kneeling or stooping
SkeletalMuscle Index^†^	1.03 (0.87, 1.21)	0.89 (0.74, 1.07)	0.88 (0.77, 1.00)	0.96 (0.84, 1.10)	1.08 (0.86, 1.35)	1.04 (0.94, 1.14)	0.92 (0.80, 1.07)	0.93 (0.79, 1.10)	1.01 (0.91, 1.12)
SkeletalMuscle Density^†^	0.88 (0.76, 1.02)	0.98 (0.83, 1.15)	0.83* (0.75, 0.92)	0.84** (0.73, 0.96)	0.85 (0.70, 1.03)	0.91* (0.85, 0.99)	0.84** (0.76, 0.94)	0.77** (0.67, 0.90)	0.87** (0.79, 0.95)
Skeletal Muscle Gauge^††^	0.95 (0.89, 1.01)	0.98 (0.91, 1.05)	0.92** (0.88, 0.96)	0.94* (0.89, 0.99)	0.95 (0.87, 1.03)	0.97 (0.94, 1.01)	0.91** (0.87, 0.96)	0.91** (0.85, 0.97)	0.95* (0.91, 0.99)

^*^ *p* = < 0.05 ^*^p = < 0.01.

^†^Relative risks for SMI and SMD are for each 5 unit increase and not single units.

^††^Relative risks for SMG are for each 100 point increase and not single units.

Abbreviations: SMI, Skeletal Muscle Index; SMD, Skeletal Muscle Density; SMG, Skeletal Muscle Gauge; KPS, Karnofsky Performance Status; TUG, Timed Up and Go; IADL, Instrumental Activities of Daily Living.

**Figure 1:**
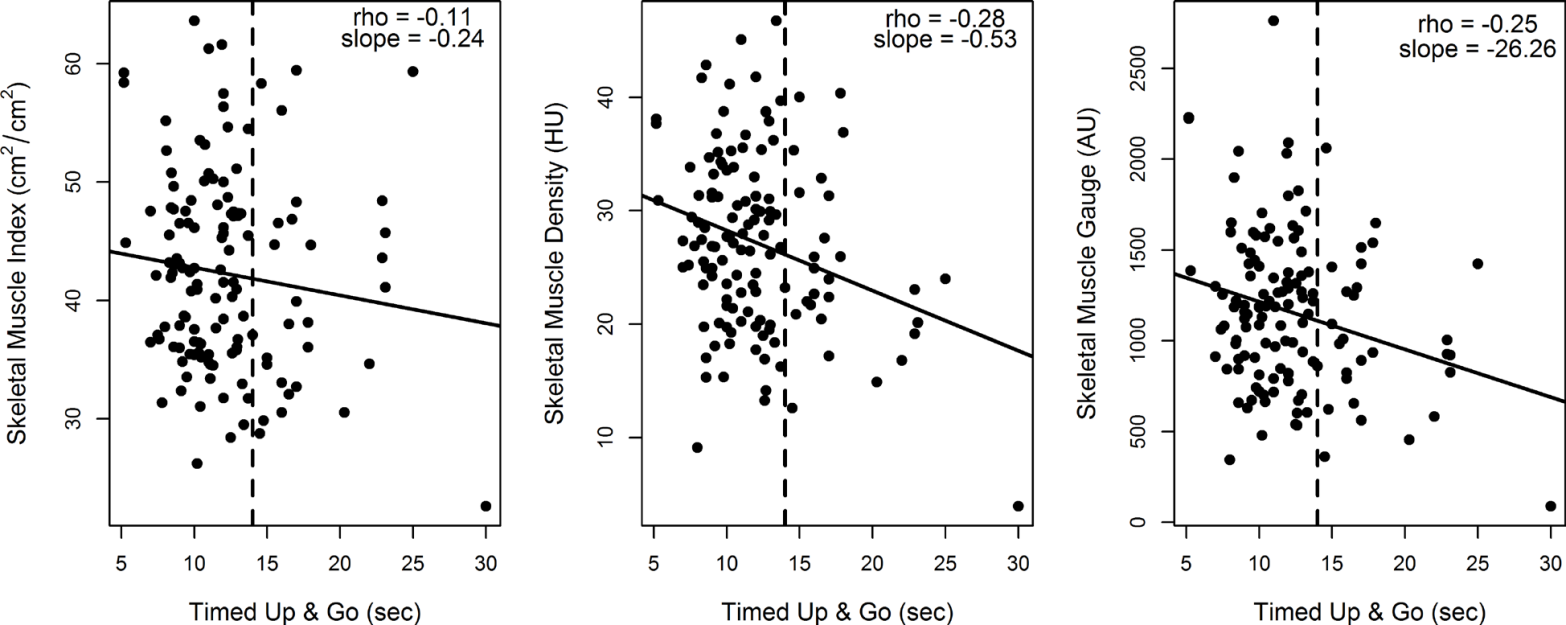
Correlation of skeletal muscle measures and Timed Up and Go (*N* = 132) Illustrates the correlation of skeletal muscle measures with performance on the Timed Up and Go (TUG) physical performance assessment. Skeletal muscle index has a very weak negative correlation with TUG scores (−0.11) while skeletal muscle density and skeletal muscle gauge have a moderate negative correlation (−0.28 and −0.25, respectively). We limited this analysis to individuals that were able to complete the TUG (*n* = 132) and excluded those that were either unable to complete (*n* = 51) or were missing the score (*n* = 2).

As the TUG is a well validated objective measure of physical performance [13], we chose to further explore this measure as a continuous variable. We limited this analysis to individuals that were able to complete the TUG (*n* = 132) and excluded those that were either unable to complete (*n* = 51) or were missing the score (*n* = 2). SMI had a weak negative correlation with TUG completion times (rho = −0.11, slope = −0.24, *p* = 0.19) (See Figure 1). Both SMD and SMG had moderate negative correlations with TUG completion times (rho = −0.28, *p* = 0.001 and rho = −0.25, *p* = 0.001, respectively). We also explored imputed the results of participants unable to complete the TUG assessment as prolonged (at 30 seconds), and this did not substantially alter the correlations. The predicted SMI, SMD, and SMG for a TUG time of 14 seconds were 41.8 cm^2^m^2^, 26.1HU, and 1110.5 AU respectively.

## DISCUSSION

Low skeletal muscle mass, referred to as sarcopenia within oncology, has been associated with adverse outcomes including treatment-related toxicity and death in patients with cancer. In our sample of older adults with cancer undergoing GA, contrary to our original hypothesis we found skeletal muscle mass was not associated with physical function or impairments in physical function. Furthermore, using cut-points commonly reported in the oncology literature, we also found no association with physical function impairments. Rather, skeletal muscle attenuation (represented as SMD), which reflects muscle lipid content, was more associated with physical function than just muscle quantity. This suggests that focusing on SMI alone for a definition of sarcopenia, as most publications within oncology have to date, misses critical information regarding physical function and performance. Commonly used definitions of sarcopenia in oncology research based on muscle mass alone poorly correspond to the consensus definition of sarcopenia used more broadly within the literature [5, 8].

Although contrary to our original hypothesis, our results are in line with others outside of the oncology field. The Health, Aging and Body Composition (Health ABC) study was designed to prospectively determine the role of changes in body composition in community dwelling older adults and represents one of the largest prospective studies to date exploring body composition. In their study of 3,075 patients, they showed that muscle attenuation and muscle strength were the only measures that independently predicted physical function [14]. They also found that reduced muscle attenuation was independently associated with poor lower extremity performance [15]. Although muscle mass does play a role in strength and performance, changes in lean mass only explain a small portion of variability of strength decline (~5%) and attenuation values of muscle on CT appear to account for differences in muscle strength not attributed to muscle quantity [6, 16]. The Health ABC study ultimately concluded that strength, as a marker of muscle quality, and not muscle mass (i.e. quantity), was most important in estimating mortality [17].

The field of oncology has examined body composition and sarcopenia through a different lens. Cancer cachexia is a multifactorial syndrome caused by a combination of elevated inflammatory response, alterations in metabolism, and reduced food intake [18]. Cancer associated skeletal muscle wasting is likely caused by a multitude of factors, some similar and some dissimilar, compared to skeletal muscle loss in non-cancer populations [18]. There is a clear and consistent association of skeletal muscle loss with poorer outcomes in cancer; including increased chemotherapy toxicity and reduced overall survival [1, 19]. However, a focus purely on skeletal muscle quantity omits important information and does not appear to tell the whole story. Oncologic studies examining skeletal muscle should ideally incorporate several metrics of skeletal muscle to fully appreciate and assess the complexity of sarcopenia and better understand its relationship to important cancer-specific outcomes. As the term sarcopenia has now been defined with its own ICD10 code (M62.84) to include both decreased muscle mass and weakness that results in functional problems, *myopenia* or the more general term *muscle wasting* may be a better term to replace sarcopenia when focusing solely on clinically relevant muscle loss [10, 11]. Myopenia specifically describes clinically relevant muscle loss or wasting that is associated with increased risk of morbidity or mortality [11].

Our study has several limitations. As our data was cross-sectional, we cannot address whether there is a causal relationship between physical function and fat infiltration in muscle. Whether the presence of fatty infiltration of muscle directly affects muscle contractility, muscle fiber recruitment, or muscle metabolism, thereby affecting muscle function, remains unknown. As our study sample included a variety of cancer types across various time points in the cancer care continuum, we are not able to draw any conclusions regarding the impact of cancer and its treatment on skeletal muscle or physical function. More specifically, about half of our sample was actively receiving oncologic treatment and it is well-known that cancer treatments can impact physical function [20]; however, it is uncertain how this may have impacted our correlations between physical function and CT imaging. Our study was meant to be an exploratory cross-sectional study across all cancer types, and clearly more work is needed to understand the impact of specific cancers and treatments on body composition. We measured physical function as part of a routine GA and do not have direct measures of muscle strength (such as grip strength or knee flexor) that would have provided additional detail. Therefore, we reported muscle “quality” only per mean muscle density and were not able to calculate strength per unit of muscle mass.

In summary, we have shown that skeletal muscle mass obtained from routine CT imaging was not associated with physical function impairments and was poorly correlated with physical performance in our sample of older adults with cancer. The ‘quality’ and composition of skeletal muscle, rather than the quantity, is likely more important in identifying and understanding impairments in physical function. Although traditional cutpoints used for defining sarcopenia in oncology are associated with increased mortality, they are poorly correlated with physical function and not consistent with the broader definition of sarcopenia. We suggest that studies that focus solely on muscle loss or wasting use the term myopenia or the more general term “muscle wasting disease” rather than sarcopenia [10, 11]. Further prospective work is necessary to adequately define and understand sarcopenia in adults with cancer that includes skeletal muscle metrics, muscle strength, and physical function. Until that time, incorporating skeletal muscle attenuation into CT imaging based studies as a surrogate of muscle composition may be beneficial to better understand physical function and performance. Using an integrated measurement of both attenuation and mass, such as SMG, may represent a promising integration of these two important variables that requires further exploration. CT imaging based body composition measures represent a unique and potentially promising opportunity to identify adults with cancer at risk for poor outcomes and may be useful in targeting future interventions.

## MATERIALS AND METHODS

### Participants

The sample for this study was derived from the Carolina Seniors Registry (CSR) (NCT01137825). The CSR was developed in 2009 as a large observational cancer registry to collect geriatric assessment (GA) data on older adults (≥ 65) with cancer. The CSR eligibility includes any patient age 65 or greater with a diagnosis of cancer. The only exclusion criteria includes the ability to speak and read English, as the GA questionnaires and assessments are not validated in other languages. As the CSR is predominately a research tool, the GAs were performed by our research staff. For a more detailed description of the CSR including the sampling methods, recruiting procedures, and performance of assessments, please see Williams *et al*. [21]. Using the CSR and electronic medical records, we identified patients recruited at the outpatient clinics of the North Carolina Cancer Hospital with any available CT abdominal imaging within 60 days of completing the GA. Of the 771 patients available with the CSR, 207 had abdominal CT imaging within 60 days of the completion of GA measures, and 185 with CT images adequate for body composition evaluation. This study was approved by the Institutional Review Board of the University of North Carolina (IRB #15–1524).

### Physical function measures

The CSR utilizes a validated GA tool designed specifically for use in older adults with cancer [21, 22]. The GA is comprised of both a health-care professional portion and patient reported measures. For the purposes of this study, we restricted our focus to the physical function domain within the GA. The health-care provider assessed function through the Timed Up and Go (TUG) test [13] and the Karnofsky Performance Status (KPS) scale [23] and patient-reported measures of instrumental activities of daily living (IADL) [24], self-reported falls [25], and physical health [26]. The KPS is a global composite measure that assesses the ability to perform normal activity and the amount of assistance required. The KPS scale generally ranges from 20 (“very sick, hospitalization required) to 100 (normal, no complaints) and was rated by the research staff at the time of performing the GA [23]. The TUG test asks the participant to stand from a chair, walk a distance of approximately 10 feet, turn around, walk back to their chair, and sit-down. The total seconds required to complete the test is recorded or “inability to complete” is noted if a participant is unable to complete the test. The GA utilizes the physical health questionnaire from the Medical Outcomes Survey that inquiries about limitations in engaging in various physical activities [26]. Cut-points were used to define impairments for each respective scale or measure based on the support literature as previously published [27]. These include a KPS < 80 [23], the presence of 1 or more falls [27], a TUG test greater than 14 seconds [13], and any reported impairment or limitation in IADL or physical health activity [24, 26].

### CT-based body composition analysis

Abdominal CT images were acquired from the UNC Picture Archiving and Communication office. Using Impac radiological software (Mountain View, CA), transverse sections at the L3 vertebral level were identified and extracted for external analysis. Automated image segmentation software was then used to analyze the L3 lumbar segments [28, 29]. The software recognizes muscle tissue based on density thresholds between −29 and +150 Hounsfield Units (HU) and provides an unbiased estimation of the cross-sectional skeletal muscle area. Images were then reviewed and corrected for accuracy and verified by two authors (GRW, SSS). The measured skeletal muscle area was then normalized for height (in meters) to calculate a skeletal muscle index (SMI) (cm^2^/m^2^) using the following formula: (skeletal muscle area-cm^2^)/ (patient height-m^2^). Skeletal muscle density (SMD) was derived by averaging the HU of skeletal muscle of the cross-sectional image. The attenuation of skeletal muscle is a non-invasive radiological technique to indirectly assess muscle fat content, known as myosteatosis. The density of skeletal muscle is inversely related to muscle fat content [30].

To integrate both the quantity (SMI) and attenuation (SMD), we generated the skeletal muscle gauge (SMG) by multiplying SMI x SMD. The actual units for SMG are (cm^2^ tissue area x average HU)/ (m^2^ height) and for simplicity we chose to represent as arbitrary units (AU). This method was first introduced by Weinberg *et al*. and showed better correlation with aging than either SMD or SMI alone [31]. SMG has also been associated with toxicity and survival in adults with metastatic breast cancer receiving taxane therapy [32].

### Statistical analysis

Descriptive statistics summarize the baseline characteristics of the sample, and independent *t*-tests are used to compare differences in skeletal muscle measures between the individual physical function variables. Given the known significant differences by gender in body composition metrics, we controlled for sex in the log-binomial regression models. Relative risks based on increasing levels of the continuous body composition measures are reported, along with 95% confidence intervals. Secondary models were fit adjusting for sex, age, treatment phase, and BMI, and model fit was evaluated by comparing the area under the receiver operating curve (AUC). Pearson correlation coefficients are reported for the relationship between continuous TUG completion scores and skeletal muscle measures, and linear regression was used to estimate the slope of the best fit line. SAS statistical software version 9.4 (SAS Institute Inc., Cary, NC) was used for all analyses.

## References

[1] Shachar SS, Williams GR, Muss HB, Nishijima TF (2016). Prognostic value of sarcopenia in adults with solid tumours: A meta-analysis and systematic review. Eur J Cancer.

[2] Rier HN, Jager A, Sleijfer S, Maier AB, Levin MD (2016). The prevalence and prognostic value of low muscle mass in cancer patients: a review of the literature. Oncologist.

[3] Mourtzakis M, Prado CMM, Lieffers JR, Reiman T, McCargar LJ, Baracos VE (2008). A practical and precise approach to quantification of body composition in cancer patients using computed tomography images acquired during routine care. Applied Physiology Nutrition and Metabolism-Physiologie Appliquee Nutrition Et Metabolisme.

[4] Baumgartner RN, Koehler KM, Gallagher D, Romero L, Heymsfield SB, Ross RR, Garry PJ, Lindeman RD (1998). Epidemiology of sarcopenia among the elderly in New Mexico. American Journal of Epidemiology.

[5] Cruz-Jentoft AJ, Baeyens JP, Bauer JM, Boirie Y, Cederholm T, Landi F, Martin FC, Michel JP, Rolland Y, Schneider SM, Topinkova E, Vandewoude M, Zamboni M (2010). Sarcopenia: European consensus on definition and diagnosis: Report of the European Working Group on Sarcopenia in Older People. Age Ageing.

[6] Goodpaster BH, Park SW, Harris TB, Kritchevsky SB, Nevitt M, Schwartz AV, Simonsick EM, Tylavsky FA, Visser M, Newman AB (2006). The loss of skeletal muscle strength, mass, and quality in older adults: the health, aging and body composition study. J Gerontol A Biol Sci Med Sci.

[7] Delmonico MJ, Harris TB, Lee JS, Visser M, Nevitt M, Kritchevsky SB, Tylavsky FA, Newman AB, Health A, Body S (2007). Composition, Alternative definitions of sarcopenia, lower extremity performance, and functional impairment with aging in older men and women. J Am Geriatr Soc.

[8] Studenski SA, Peters KW, Alley DE, Cawthon PM, McLean RR, Harris TB, Ferrucci L, Guralnik JM, Fragala MS, Kenny AM, Kiel DP, Kritchevsky SB, Shardell MD (2014). The FNIH sarcopenia project: rationale, study description, conference recommendations, and final estimates. J Gerontol A Biol Sci Med Sci.

[9] Fielding RA, Vellas B, Evans WJ, Bhasin S, Morley JE, Newman AB, Abellan van Kan G, Andrieu S, Bauer J, Breuille D, Cederholm T, Chandler J, De Meynard C, Donini L (2011). Sarcopenia: an undiagnosed condition in older adults. Current consensus definition: prevalence, etiology, and consequences. International working group on sarcopenia. J Am Med Dir Assoc.

[10] Anker SD, Coats AJ, Morley JE, Rosano G, Bernabei R, von Haehling S, Kalantar-Zadeh K (2014). Muscle wasting disease: a proposal for a new disease classification. J Cachexia Sarcopenia Muscle.

[11] Fearon K, Evans WJ, Anker SD (2011). Myopenia-a new universal term for muscle wasting. J Cachexia Sarcopenia Muscle.

[12] Martin L, Birdsell L, Macdonald N, Reiman T, Clandinin MT, McCargar LJ, Murphy R, Ghosh S, Sawyer MB, Baracos VE (2013). Cancer cachexia in the age of obesity: skeletal muscle depletion is a powerful prognostic factor, independent of body mass index. J Clin Oncol.

[13] Podsiadlo D, Richardson S (1991). The timed up and go - a test of basic functional mobility for frail elderly persons. J Am Geriatr Soc.

[14] Visser M, Goodpaster BH, Kritchevsky SB, Newman AB, Nevitt M, Rubin SM, Simonsick EM, Harris TB (2005). Muscle mass, muscle strength, and muscle fat infiltration as predictors of incident mobility limitations in well-functioning older persons. J Gerontol A Biol Sci Med Sci.

[15] Visser M, Kritchevsky SB, Goodpaster BH, Newman AB, Nevitt M, Stamm E, Harris TB (2002). Leg muscle mass and composition in relation to lower extremity performance in men and women aged 70 to 79: the health, aging and body composition study. J Am Geriatr Soc.

[16] Goodpaster BH, Carlson CL, Visser M, Kelley DE, Scherzinger A, Harris TB, Stamm E, Newman AB (2001). Attenuation of skeletal muscle and strength in the elderly: The Health ABC Study. J Appl Physiol.

[17] Newman AB, Kupelian V, Visser M, Simonsick EM, Goodpaster BH, Kritchevsky SB, Tylavsky FA, Rubin SM, Harris TB (2006). Strength, but not muscle mass, is associated with mortality in the health, aging and body composition study cohort. J Gerontol A Biol Sci Med Sci.

[18] Fearon K, Arends J, Baracos V (2013). Understanding the mechanisms and treatment options in cancer cachexia. Nature Reviews Clinical Oncology.

[19] Kazemi-Bajestani SM, Mazurak VC, Baracos V (2016). Computed tomography-defined muscle and fat wasting are associated with cancer clinical outcomes. Semin Cell Dev Biol.

[20] Stein KD, Syrjala KL, Andrykowski MA (2008). Physical and psychological long-term and late effects of cancer. Cancer.

[21] Williams GR, Deal AM, Jolly TA, Alston SM, Gordon BB, Dixon SA, Olajide OA, Chris Taylor W, Messino MJ, Muss HB (2014). Feasibility of geriatric assessment in community oncology clinics. J Geriatr Oncol.

[22] Hurria A, Gupta S, Zauderer M, Zuckerman EL, Cohen HJ, Muss H, Rodin M, Panageas KS, Holland JC, Saltz L, Kris MG, Noy A, Gomez J (2005). Developing a cancer-specific geriatric assessment: a feasibility study. Cancer.

[23] Karnofsky D, Burchenal J (1948). The clinical evaluation of chemotherapeutic agents in cancer, in macleod CM: evaluation of chemotherapeutic agents.

[24] Fillenbaum GG, Smyer MA (1981). The development, validity, and reliability of the oars multidimensional functional assessment questionnaire. Journals of Gerontology.

[25] Naeim A, Reuben D (2001). Geriatric syndromes and assessment in older cancer patients. Oncology (Williston Park).

[26] Stewart AL, Ware JE (1992). Measuring Functioning and Well-Being: The Medical Outcomes Study Approach.

[27] Jolly TA, Deal AM, Nyrop KA, Williams GR, Pergolotti M, Wood WA, Alston SM, Gordon BB, Dixon SA, Moore SG, Taylor WC, Messino M, Muss HB (2015). Geriatric assessment-identified deficits in older cancer patients with normal performance status. Oncologist.

[28] Chung H, Cobzas D, Birdsell L, Lieffers J, Baracos V (2009). Automated segmentation of muscle and adipose tissue on CT images for human body composition analysis. in SPIE Medical Imaging. International Society for Optics and Photonics.

[29] Popuri K, Cobzas D, Esfandiari N, Baracos V, Jagersand M (2016). Body composition assessment in axial CT images using FEM-based automatic segmentation of skeletal muscle. IEEE Trans Med Imaging.

[30] Aubrey J, Esfandiari N, Baracos VE, Buteau FA, Frenette J, Putman CT, Mazurak VC (2014). Measurement of skeletal muscle radiation attenuation and basis of its biological variation. Acta Physiol (Oxf).

[31] Weinberg M, Shachar S, Deal A, Williams G, Nyrop K, Alston S, Muss H (2016). Characterization of skeletal muscle and body mass indices in younger and older women with stage II and III breast cancer. J Am Geriatr Soc.

[32] Strulov Shachar S, Deal AM, Weinberg M, Nyrop KA, Williams GR, Nishijima TF, Benbow JM, Muss HB (2017). Skeletal muscle measures as predictors of toxicity, hospitalization, and survival in patients with metastatic breast cancer receiving taxane based chemotherapy. Clin Cancer Res.

